# Phytochemical Analysis, Antioxidant Activity, Antimicrobial Activity, and Cytotoxicity of *Chaptalia nutans* Leaves

**DOI:** 10.1155/2020/3260745

**Published:** 2020-05-01

**Authors:** Letiele Bruck de Souza, Amanda Leitão Gindri, Thainara de Andrade Fortes, Thais Felli Kubiça, Jefferson Enderle, Rafael Roehrs, Sidnei Moura e Silva, Vanusa Manfredini, Elton Luís Gasparotto Denardin

**Affiliations:** ^1^Laboratório Estudos Físico–Químico e Produtos Naturais (LEFQPN), Federal University of Pampa (UNIPAMPA), P. Box 118, Uruguaiana, RS 97501-970, Brazil; ^2^Laboratório de Plantas Medicinais, Universidade Regional Integrada do Alto Uruguai e Das Missões, Santiago, RS, Brazil; ^3^Universidade de Caxias do Sul (UCS), Caxias do Sul, RS, Brazil; ^4^Grupo de Estudos em Estresse Oxidativo (GESTOX), Federal University of Pampa (UNIPAMPA), Uruguaiana, RS, Brazil

## Abstract

**Objective:**

This work was to evaluate the chemical constitution of the hydromethanolic (30/70 methanol-water) macerating extract obtained from the leaves of *C. nutans*, as well as to study the antioxidant, antimicrobial, cytotoxic, and genotoxic activity of the species. *Materials and methods*. Phytochemical screening, antioxidant activity (total phenolic, total flavonoid, condensed tannins content, DPPH radical, and FRAP), antibacterial activity (*P. aeruginosa, B. cereus, E. epidermidis, E. coli, S. aureus, E. faecalis, P. mirabilis, Candida glabrata* (clinical isolate), *Candida tropicalis* (clinical isolate), *C. krusei* (clinical isolate), *and C. albicans* (clinical isolate)), and oxidative stress parameters (TBARS, carbonyl protein, and DCFH) were analyzed according to the literature. Toxicity of *C. nutans* was evaluated using an alternative method, *D. melanogaster*, as well as a locomotor assay.

**Results:**

The phytochemical screening test of methanolic leaves extract revealed the presence of alkaloids, coumarins, quaternary bases, phenolics, flavonoids, tannins, and free steroids. A quantitative phytochemical study indicated the total phenol (30.17 ± 1.44 mg/g), flavonoid (21.64 ± 0.66 mg/g), and condensed tannins (9.58 ± 0.99 mg/g). DPPH (345.41 ± 5.35 *μ*g/mL) and FRAP (379.98 ± 39.25 *μ*M FeSO4/mg sample) show to extract of *C. nutans* leaves an intermediate value, indicating moderate antioxidant activity of the extract. Antibacterial results revealed only a positive result (antimicrobial activity) for the hexane fraction which significantly inhibited the microorganisms *E. epidermidis, C. tropicalis, C. glabrata*, and *C. krusei* at a concentration of 1000 *μ*g/mL. TBARS, carbonyl protein, and DCFH demonstrate that the extract has the ability to protect the cell from protein and lipid damage, as well as the inhibition of oxygen-derived radicals at the three concentrations tested: 0.1, 1, and 10 mg/mL. Regarding the toxicity of *C. nutans* extract against *D. melanogaster*, it was found that until the concentration of 15 mg/mL, the extract showed no toxicity and that the LC_50_ obtained was 24 mg/mL. Results show that the *C. nutans* extract leaves used to prevent PQ damage were effective in reducing flies' mortality and improving locomotor capacity.

**Conclusion:**

Our studies demonstrated for the first time that *C. nutans* crude leaf extract has high antioxidant capacity both in vitro and in vivo through different analysis techniques. These results make it possible to infer future applications in the pharmacological area, evidenced by the low toxicity observed in D. melanogatser, as well as the ability to neutralize different sources of RONS.

## 1. Introduction

The interest of the scientific class in the study of compounds of plant origin is increasing worldwide, especially in developing countries where the use of herbal medicines is widely used for their basic health needs [1].

It is known that medicinal plants have been used worldwide since ancient times for the treatment of various diseases, including asthma, abdominal disorders, skin diseases, respiratory and urinary complications, and liver and cardiovascular disease [2, 3]. This empirical knowledge comes from the plant defense system, which generates numerous compounds with diverse molecular structures, far superior to those derived from synthetic products [4], so the great interest in the elucidation of new active principles.

Only in the last two decades, studies focused on natural compounds with antioxidant activities have shown enormous growth, since a substantial amount of evidence has indicated that cell damage caused by oxidative stress has been considered an important factor in aging and in the development of a wide variety of pathologies, such as autoimmune diseases, infectious and/or inflammatory diseases, and degenerative and neurodegenerative diseases [5, 6]. Thus, the importance of the search for natural products with antioxidant effect is emphasized, as they are able to prevent,

stabilize, or disable free radicals before they attack biological targets in cells (DNA, proteins, and lipids) [7].

Often, people use plants to treat a variety of diseases, without knowing their toxic potential, which can be harmful to human health. One of the main problems in the use of natural products is the belief that products of plant origin are free from adverse reactions and toxic effects [8]. Thus, studies on the toxicity of medicinal plants are important, in order to define the risk associated with phytotherapy, as well as guide research for the isolation of certain compounds until the development of new drugs.

The species *Chaptalia nutans* (L.) Pol. (*C. nutans)*, belonging to the family Asteraceae, known as “língua-de-vaca” or “arnica-do-campo,” is an annual herbaceous species native to the Americas and can be found from Mexico to Argentina [9, 10, 11]. The species is easily distinguished by having herbaceous size, blackened roots, very small stem from which sessile, papyraceous, lyrical, rosy, and hairy leaves emerge on the back, and long thin floral scents of up to 79 cm (length), which support the inflorescences [12, 13, 14]. The leaf has a unistratified epidermis covered by a clearly streaked cuticle and numerous trichomes on the back, these characteristics being used for taxonomic purposes and also in drug morphodiagnosis [15].

Widely used in folk medicine, its leaves are indicated internally as laxative and anticough medications, especially in the traumatisms, wounds, and hemorrhages in topical preparations [12]. Pharmacological assays were conducted with leaves of C. *nutans*, in order to justify the effects attributed to them, and the anti-inflammatory, cholinergic. and antimicrobial activities were proven [16, 17]. From the crude extract of the roots of this plant, a coumarin, 7-O-*β*-D-glucopyranosylnutanocoumarine, with antibacterial activity for *Bacillus subtilis* and *Staphylococcus aureus* was found[18, 19]. According to the same authors, the healing of contaminated wounds comes from this compound.

Although these reports validate its folk use, to date, no studies of its phytochemical composition, antioxidant activity, and cytotoxicity have been evidenced, thus making important new investigations. Interest in the demand for more plant-derived drugs is gradually increasing, which are sometimes considered safe when compared to synthetic drugs [20].

The objective of this work was to evaluate the chemical constitution of the hydromethanolic (30/70 methanol-water) macerating extract obtained from the leaves and roots of *C. nutans*, as well as to study the antioxidant, antimicrobial, cytotoxic, and genotoxic activity of the species.

## 2. Materials and Methods

### 2.1. Plant Material

*C. nutans* species were collected manually from São Francisco de Assis-Rio Grande do Sul (Brazil) (Lat.: 29°33′01″S *e* Long. 55°07′52″W). The plant material was identified by Patrícia de Oliveira Neves (Biologist), Federal University of Pampa (Campus São Gabriel), Brazil. A voucher specimen was deposited in the Herbarium of Federal University of Pampa (HBEI 203).

Leaf (18.19 g) material was dried, powered, and extracted using hydromethanolic (30/70 methanol-water) macerating (30 g/100 mL) over four weeks. After that, the crude extract was concentrated using a rotary evaporator. For antibacterial assay, the crude extract was subjected to partitioning by sequential extraction using increasing polarity solvents: hexane (Hex), chloroform (CHCl_3_), ethyl acetate (EtOAc), and n-butanol (BuOH). The fractions were concentrated in a rotary evaporator.

### 2.2. Phytochemical Analysis

The plant extract was assessed for the existence of cyanogenic glycosides, phenols, tannins, anthocyanins, proanthocyanidins, flavonoids, catechins, steroids, triterpenoids, saponins, resins, alkaloids, and quaternary bases by the phytochemical analysis (screening) using typical standard methods [21].

#### 2.2.1. Total Polyphenols, Flavonoids, and Condensed Tannins Assay

The total phenolic compounds in the leaf extract were determined according to the Folin–Ciocalteu method [22]. Results of total phenolic contents were expressed as milligrams of gallic acid equivalents (GAE) per mL extract.

Total flavonoid compound was measured by the aluminum chloride colorimetric assay based on the work by Woisky and Salatino [23]. Total flavonoid compound of the extract was expressed as mg quercetin equivalent/mL extract (mg QUE/mL EXT).

Condensed Tannins were determined by Morrison et al. [24]. Briefly, 0.1 mL of the leaf sample was added to aliquots (25 mg/mL) to 0.9 mL of methanol and 5.0 mL of vanillin reagent. Then, it was heated in a water bath at 40°C for 20 minutes, and absorbance was read at 500 nm. The analysis was performed in triplicate using catechin as standard (0.0025–0.2 mg·mL^−1^) (*Y* = 0.0015*x* − 0.0005, *r* = 0.9968). The results were expressed in milligram equivalents of catechin per milliliter of extract/fraction (mg CAT/mL EXT).

#### 2.2.2. Determination of Chemical Composition by HPLC-DAD-MS

The identification and quantification of the secondary metabolites of *C. nutans* crude extract followed the methodology proposed by Vieira et al. [25], with minor modifications. High-performance liquid chromatography coupled with a mass spectrometry detector (HPLC-DAD-MS) corresponded to a Shimadzu Prominence UFLC (Shimadzu, Kyoto, Japan) equipped with an Auto-Sampler (SIL-20AHT), two Shimadzu LC-20ADT reciprocating pumps connected to the degasser DGU20A3R, integrator CBM20A, UV–VIS detector DAD SPD-M20A, and oven CTO-20A.

The HPLC system was coupled to the compact quadrupole time-of-flight (Q-TOF) mass analyzer (Bruker Daltonik GmbH, Bremen, Germany), which was controlled using Ot of Control Software. The parameters for analysis were set using negative ion modes with spectra acquired over a large range from 50 to 1200 *m*/*z*. The optimum values of the ESI-MS parameters were a capillary voltage of 4500 V, drying gas temperature of 215°C, drying gas flow of 10.0 L/min, nebulizing gas pressure of 5.0 Bar, collision RF of 150 Vpp, transfer time of 70 ls, and a prepulse storage of 5 ls. Additionally, automatic MS/MS experiments were performed using nitrogen as collision gas and by adjusting the collision energy values as follows: *m*/*z* 100, 20 eV; *m*/*z* 500, 30 eV; and *m*/*z* 1000, 35 eV. The MS data were analyzed using Data Analysis 4.0 software (Bruker Daltonics, Bremen, Germany).

Analyses were carried out within the C-18 column (4.6 mm × 250 mm, Merck, Germany) packed with 5 *μ*m diameter particles and within the C-18 precolumn (RP 18 5 *μ*m, Merck, Germany). The first mobile phase, phase A, was two percent acetic acid at a pH of 4.2. The second mobile phase, phase B, used methanol, acetic acid, and distilled water at a ratio of 18 : 1:1, respectively. The gradient elution was 0 min: 20% of B, 0–25 min: 50% of B, 25 min: 20% of B, and 30 min: 20% of B (end of run), at the flow rate of 0.8 mL·min^−1^. The peaks were identified by comparing the present results with the retention times and mass spectrums from the software library and external standards. The external standards included a 40% isoflavone pool (Daidzin: 3.1%, Glycitin: 1.56%, Genistin: 0.98%, Daidzein: 35.49%, Glycitein: 0.1%, and Genistein: 0.03% - Dongming Huiren Biological Products, Shandong, China) between 0.156 and 2.34 mg/mL of glycitin, 3.5–53.2 mg/mL of daidzein, 3–4.5 *μ*g/mL of genistein, and levels of caffeic acid, gallic acid, clorogenic acid, catequin, luteolin, cumarin, quercetin, and rutin (all standards by Sigma-Aldrich, St Louis, MO, USA) between 1.5 and 24 *μ*g/mL. The samples and standards were tested in triplicate. The results are presented as mean ± standard deviation.

### 2.3. In Vitro Antioxidant Activity

#### 2.3.1. DPPH Radical Scavenging Activity

The percentage antioxidant activity (AA%) of the leaf extract was obtained using the DPPH (2,2-diphenyl-1-picryl-hydrazyl) radical absorbance assay, according to the procedure described by Choi et al. [26] with some changes. The reaction mixture contained sample and DPPH in ethanol at different concentrations (250, 125, 62.5, 31.25, 15.62 *e* 7.81 *μ*g/mL). When DPPH reacts with an antioxidant compound, which can donate hydrogen, it is reduced. The positive control was ascorbic acid at the same sample concentration. The changes in color (from deep violet to light yellow) were read (Absorbance (Abs)) at 518 nm after 30 min of reaction using a UV-VIS spectrophotometer (Spectrophotometer Pharo 100 Spectroquant®-Merck KGaA, Darmstadt, Germany). The reaction occurred in 30 minutes, and soon after that the absorbance was read in the spectrophotometer at 518 nm. The whole test was performed in triplicate. The percent DPPH˙ scavenging effect was calculated from the following equation:

(1) $$\begin{array}{c} \text{DPPH}\left(\text{scavenging effect }\%\right)=100-\frac{{\text{Abs}}_{\text{sample}}-{\text{Abs}}_{\text{blank}}}{{\text{Abs}}_{\text{contol}}}\times 100,\; \end{array}$$

where Abs _sample_, Abs _blank_, and Abs _control_ are the absorbance of the sample, blank, and negative control. The inhibitory concentration IC_50_ was calculated by interpolation from linear regression analysis.

#### 2.3.2. Ferric Reducing Antioxidant Power Assay (FRAP)

The antioxidant power activity of C*. nutans* performed by the iron reduction ability (FRAP) was performed according to the methodology described by Rufino et al. [27], with minor modifications. The samples were prepared at the concentration of 1000 *μ*g/mL and diluted in distilled water. The test was performed in triplicate, from the addition of 200 *μ*L of the sample and 1800 *μ*L of the FRAP reagent. Subsequently, the samples were stored in an oven (37°C) for four minutes. The reading was performed on an Ultraviolet-Visible (UV-VIS) spectrometer at 593 nm. A standard curve of ferrous sulfate at concentrations of 1000 mmol/L to 62.5 mmol/L (*y* = 0.000049*x* + 0.036181, *R*^2^ = 0.9922) was used to perform the calculations.

#### 2.3.3. Lipid Peroxidation Assay (TBARS)

Thiobarbituric acid reactive substances were used as a measure of oxidative stress according to Okhawa et al. [28]. In summary, the samples were mixed with 1 mL of 10% trichloroacetic acid and 1 mL of 0.67% thiobarbituric acid and then heated in a boiling water bath for 30 minutes. TBARS was determined by absorption of 535 nm. The results were expressed as malondialdehyde equivalent per milligram of protein (Eq MDA/mg protein).

#### 2.3.4. TBARS Induced by Ferrous Sulphate (FS)

Another method used for lipid peroxidation analysis was TBARS (thiobarbituric acid reactive species), according to the methodology described by VYNCKE [29], with some modifications. In this method (with damage inducer), 1% diluted egg yolk (v/v) was used in 100 mM TRIS HCl pH 7.4 buffer, using ferrous sulfate aqueous at a concentration of 13.9 *μ*g/mL as a damage inducer. The concentrations of *C. nutans* extract used were 10, 100, and 1000 *μ*g/mL^−1^ (p/v). Buffer solution was used as negative control. Absorbance was read on a 532 nanometer wavelength spectrophotometer. Analysis was performed in quadruplicate.

#### 2.3.5. Determination of the Protein Content of Carbonyl Groups

The determination of oxidized protein content (carbonyl grouping) was performed by the reaction of carbonyl groups with 2,4-dinitrophenylhydrazine (DNPH), as previously described by Levine et al. [30]. Briefly, heparinized whole blood was precipitated with 10% TCA and, after centrifugation, the pellet was treated with 1 mL of 0.2% DNPH in HCl (2 mol/L) or 1 mL HCl (2 mol/L) as a white. The samples were incubated for one hour at room temperature with shaking for 5 minutes. 200 *μ*L of TCA was then added, and the precipitated proteins were subsequently washed three times with 10% TCA, three times with ethanol/ethyl acetate 1 : 1 (v/v) and three times again with 10% TCA. The final precipitate was dissolved in 6 mol/L guanidine hydrochloride with TCA: the insoluble debris was removed by centrifugation. The concentration of carbonyl groups was calculated from the absorbance at 370 nm using 21.5 mmol·L/cm as the extinction coefficient for aliphatic hydrazones, and the results were expressed as carbonyl mmol per mg protein (mmol Carbonyl/mg Protein).

#### 2.3.6. Assessment of DCFH Oxidation

The evaluation of the oxidation of the 2,7-dichlorofluorescein (DCFH) of the crude extracts of the leaf of C. *nutans* was carried out to determine the level of intracellular generation of reactive oxygen and nitrogen species (RONS), a general index of stress oxidative according to Myrhe et al. [31]. The assay reaction mixture consisted of 150 *μ*L of 0.1 *μ*M potassium phosphate buffer (pH 7.4), 40 *μ*L of distilled water, 5 *μ*L of DCFH-DA (200 *μ*M, final concentration 5 *μ*M), and 5 *μ*L of the sample (1 : 10 dilution). The emission of DCF fluorescence resulting from the oxidation of DCFH was monitored for 10 min (30 s intervals) at 488 and 525 nm, excitation and emission wavelengths, respectively, using a SpectraMax plate reader (Molecular Devices, CA, USA). The rate of DCF formation was expressed as a percentage (% of control group).

### 2.4. Antimicrobial Activity Assay

Crude extracts and fractions were individually evaluated against *P. aeruginosa* (ATCC 9027), *B. cereus* (ATCC 33019), *E. epidermidis* (ATCC 1228), *E. coli* (ATCC 25922), *S. aureus* (ATCC 33019), *E. faecalis* (ATCC 29212), *P. mirabilis* (ATCC 25933), *Candida glabrata* (clinical isolate), *Candida tropicalis* (clinical isolate), *C. krusei* (clinical isolate), and *C. albicans* (clinical isolate). Antibacterial susceptibility testing was performed as recommended by CLSI (Clinical and Laboratory Standards Institute) document M07-A9 [32] and antifungal was in accordance with protocol M27-A3 for yeast fungi of the Clinical and Laboratory Standards Institute (CLSI) [33]. Bacterial strains were grown overnight at 35°C for 24 h on Mueller–Hinton agar and fungal strains at 35°C for 48 h or 72 h on Sabouraud Dextrose agar. Successive dilutions from 2000 to 15.6 *μ*g/mL of the extracts were prepared in 96-well microplates. For this, 200 mg/mL stock solutions in 1% DMSO were used. 90 *μ*L of this solution was transferred to the microplates, which already contained 100 *μ*L of the culture medium. To complete the final volume of 200 *μ*L, 10 *μ*L of inoculum was added (the optical density of the suspension was adjusted between 0.5 and 2.5 × 10³ Colony Forming Units (CFU)/mL according to the turbidimetric scale McFarland Standard). The plates were incubated at 37°C for 24 h for bacteria and at 35°C for 48 h for fungi. The MIC was calculated as the lowest dilution that showed complete inhibition of growth of the tested microorganism. For the bacteria, the 2,3,5-triphenyltetrazolium chloride developer was added and for the fungi it was visualized by turbidity assessment in the naked eye wells. All tests were performed in triplicate.

### 2.5. Toxicity Assay

#### 2.5.1. Toxicity to the Nauplii of *Artemia Salina*

The toxicity test with *Artemia salina* (Leach) nauplii was performed according to the methodology adapted by Silva et al [34].. The saline cysts were incubated at 30°C in artificial saline (23 g/L of sea salt and 0.7 g/L of sodium bicarbonate in distilled water). The culture was maintained under constant aeration and stirring 48 hours for hatching. Afterwards, ten nauplii were transferred to tubes containing artificial sea water, with three different concentrations of the extract of C*. nutans*: 0.1, 0.5, and 1.5 mg/mL. The test was performed in triplicate. The count of live and dead nauplii was performed after 24 hours. As a negative control, only artificial saline was used and as a positive control, and sodium lauryl sulfate was used. After counting live and dead nauplii, the LC_50_ and the confidence interval were calculated.

#### 2.5.2. Genotoxic Evaluation in Allium Cepa

For the toxicity test in A. *strain*, the methodology of Tedesco and Laughinghouse [35] with some modifications was used. Eight groups of 5 bulbs were placed to root in distilled water for 48–72 h. After that, the bulbs were treated with different concentrations of the crude extracts (0.1, 0.5, and 1.5 mg/mL) for 48 h, with a negative control of distilled water and positive Glyphosate 2%. Subsequently, the radicles were collected and fixed in ethanol-acetic acid (3 : 1) for another 24 hours. After this period, the radicles were removed from the fixative, packed in amber glass bottles containing 70% alcohol, and stored in the refrigerator until use.

To evaluate the antiproliferative potential, radicles were collected, which were hydrolyzed in 1 M hydrochloric acid for 5 minutes, after washing in distilled water and stained with 2% acetic orcein. The lamina were made by the crushing technique [36] and examined by observing the phases of the cell cycle (interphase, prophase, metaphase, anaphase and telophase) with the aid of an optical microscope with a 40X objective. 1000 cells were analyzed per bulb, totaling 5000 cells per treatment, and the mean cell number values of each of the cell cycle phases of *A. cepa* were calculated. The determination of the Mitotic Index (MI) was performed according to the following equation:

(2) $$\begin{array}{c} \text{MI }=\frac{\text{total of cells observed}\left(\text{cells in interphase}+\text{dividing cell number}\right)}{\text{number of cells in interphase}}\times \;100. \end{array}$$

Determination of the percentage of Abnormalities (AN) was performed according to equation (2):

(3) $$\begin{array}{c} \%\text{ abnormalities }=\frac{\text{abnormalities}}{\text{total cells}}\times \;100.\; \end{array}$$

### 2.6. Fly Behavior Assay

#### 2.6.1. Drosophila Stock

Wild-type *Drosophila melanogaster* was obtained from the National Species Stock Center, Bowling Green, OH, USA. Flies were reared on a standard corn flour diet with yeast granules as the protein source at constant temperature and humidity (22 ± 1°C; 60% relative humidity, respectively) and under a 12-h dark/12-h light cycle.

#### 2.6.2. Survival Rates

To determine the survival rate, the flies were exposed for 7 days to different extract concentrations (15, 20, 25, 30, and 35 mg/mL) mixed to the diet and evaluated by counting the number of live flies daily until the end of the trial period. For each group, 80 flies were tested. At the end of the treatments, the number of dead flies was recorded and expressed as a percentage of surviving flies compared to the control (considered 100%).

#### 2.6.3. Paraquat Exposure and *C. nutans* Extract Treatment

Adult flies, 1–4-day-old, were divided into the following groups: NC: Negative Control (1% sucrose); EC: Extract Control (10 mg/mL extract leaf); and PC: Positive Control (3 mM PQ); Treatments: T1: 3 mM PQ + 1 mg/mL extract; T2: 3 mM PQ + 5 mg/mL extract; T3: 3 mM PQ + 10 mg/mL extract. Flies were exposed to treatments for 4 days, and vials containing flies were maintained in an incubator at 22 ± 1°C with 60% relative humidity and a 12-h dark/12-h light cycle) before use in assays [37]. Exposure to PQ concentration (3 mM) and different *C. nutans* extract concentrations were based on survival curves and corresponded to minimum time and concentration required to induce significant locomotor deficits and toxicity in flies.

#### 2.6.4. Negative Geotaxis Assay

Traditional adult flies climbing assays were effected using a negative geotaxis assay [37]. Flies were submitted under brief ice anesthesia and were placed in a vertical empty plastic jar (length 15 cm, diameter 2 cm/10 flies each). After recovery from cold exposure (approximately 10 min), flies were gently tapped to the bottom of the column. The number of flies that climbed from the bottom to 15 cm mark in 8 s was counted. The assay was repeated six times, and data were expressed as the means of six trials per replicate. Similar steps were followed to score for controls also.

### 2.7. Statistical Analysis

Phytochemical analyses and *in vitro* antioxidant activity results are reported as means ± SD. In vivo and ex vivo results are expressed as means ± SEM. All experiments were performed in triplicate. Multiple comparisons were performed using one-way ANOVA followed by Bonferroni's Multiple Comparison Test, and differences were considered significant when *p* < 0.05, 0.01, and 0.001. Statistical analyses were performed using GraphPad Prism5 software.

## 3. Results

### 3.1. Phytochemical Analysis

The qualitative phytochemical analysis of the C. *nutans* species exhibited the presence of alkaloids, coumarins, quaternary bases, phenolics, flavonoids, tannins, and free steroids. The presence of these active phytoconstituents clearly demonstrated that leaf C. *nutans* have prominent antioxidant properties and a source for further exploration in pharmacological activity.

#### 3.1.1. HPLC- DAD-MS Assay

Results obtained by HPLC-DAD-MS analysis show six possible compounds present in the crude extract of *C. nutans* leaf (Table 1). The peaks were identified by comparing the results obtained with retention times and mass spectra of the software library and external standards.

#### 3.1.2. Polyphenols, Flavonoids, and Condensed Tannin Contents

Table 2A show the results obtained to polyphenols (30.17 mg/g), flavonoids (21.64 mg/g), and condensed tannins (9.58 mg/g) in methanolic extract of C. *nutans* leaves. The presence of these compounds suggests a use as an antioxidant material.

### 3.2. In Vitro Antioxidant Activity

#### 3.2.1. DPPH and FRAP Analysis

The results obtained for antioxidant activity using DPPH and FRAP analyses are show in Table 2B. The low IC_50_ observed in C. *nutans* leaf (345.41 ± 5.35 *μ*g/mL) means that this species has a high inhibition capacity of the DPPH radical with a small amount of sample. The same behavior was observed for ferric reducing antioxidant power assay (FRAP) (379.98 ± 39.25 *μ*g/mL).

#### 3.2.2. TBARS, Protein Carbonyl, and DCFH Assay

Three different concentrations of *C. nutans* extract leaf were subjected to the thiobarbituric (TBARS), protein carbonyl, and DCFH assay.

**Figure 1:**
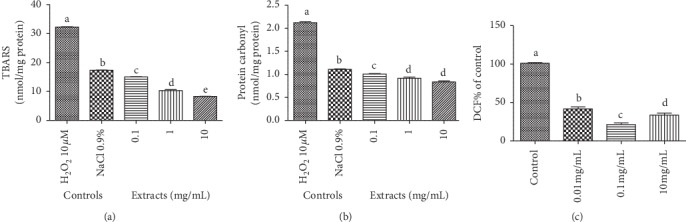
Evaluation of the antioxidant activity of the crude extract of *C. nutans* leaves by lipoperoxidation assay (TBARS) (a), carbonyl protein levels (b), and chemical deacetylation of DFCH-DA compound (c). Different letters represent statistical differences according to the Tukey test (<0.001).

As seen in Figure 1, the extract reduced the concentration of TBARS and carbonyl, as well as neutralized peroxyl radicals at the three concentrations tested (Figure 1). TBARS assay demonstrated significant differences between the results obtained for all three samples where the extract concentrations show values lower than the positive and negative controls (Figure 1(a)). It can be inferred that the C. *nutans* leaf has antioxidant activity, preventing lipid oxidation.

According to statistic results, the three extract concentrations present significant changes in protein carbonyl content as show in Figure 1(b). The leaf extract prevented the natural oxidative damage of the cells, i.e, protected the tissue from protein and lipid damage. The concentrations of 1 mg/mL and 10 mg/mL present similar results and a maximum protection of the protein tissue.

The ability to neutralize oxygen radicals was determined using DCFH assay (Figure 1(c)). Results obtained demonstrated that leaf extract have the ability to decrease activity of oxygen radical species. The three extract concentrations tested present values lower than the control, principally to the concentration of 0.1 mg/mL.

#### 3.2.3. TBARS Induced by Ferrous Sulphate (FS)

**Figure 2:**
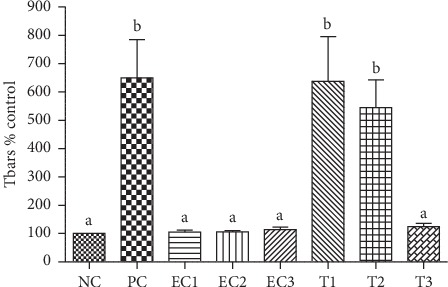
NC: Negative Control (buffer solution); PC: Positive Control (FS = Ferrous sulfate) (FS 13.9 *μ*g/mL); Extract control (EC1: Extract Control 10 *μ*g/mL; EC2: Extract Control 100 *μ*g/mL; EC3: Extract Control 1000 *μ*g/mL); and treatment (T1: FS 13.9 *μ*g/mL + extract 10 *μ*g/mL; T2: FS 13.9 *μ*g/mL + extract 100 *μ*g/mL; T3: FS 13.9 *μ*g/mL + extract 1000 *μ*g/mL). Different letters represent statistical differences according to the Tukey test (*p* < 0.0001).

In order to verify the potential of the species under study to protect lipid peroxidation induced by ferrous sulfate, egg yolk was used as lipid source. In this way, it can be observed that there was no increase in the concentration of malondialdehyde (MDA) in the sample (Figure 2), indicating that the extract does not generate lipid peroxidation at the three concentrations analyzed, not differing statistically with the negative control and between them. Regarding the treatments with induction of damage (ferrous sulfate), it was found that the extract in the lowest concentrations was not able to protect lipid peroxidation, not differing statistically from the positive control. However, for the concentration of 1000 *μ*g/mL, it was observed a decrease in MDA (promising result), demonstrating that at this concentration *C. nutans* leaf extract was able to decrease the lipid peroxidation caused by ferrous sulfate.

### 3.3. Antimicrobial Activity

Evaluation of antimicrobial activity of C. *nutans* leaf was carried out in crude extract and for hexane, chloroform, ethyl acetate, and butanol fractions. However, only positive result (antimicrobial activity) was observed for the hexane fraction (Table 3). The hexane fraction significantly inhibited the microorganisms *E. epidermidis, C. tropicalis, C. glabrata, and C. krusei* at a concentration of 1000 *μ*g/mL.

### 3.4. Toxicity and Genotoxicity

The toxicity of *C. nutans* was evaluated by the *Artemia salina* (*A. salina*) assay, analyzing the lethal concentration for 50% of the nauplii (LC_50_) (Table 4). After that, the *Allium cepa* (*A. cepa*) test was performed with previously standardized concentrations in *Artemia salina*.

The *A. salina* test demonstrated that *C.nutans* leaf presented moderate toxicity (342.89 *μ*g/mL) indicating a LC_50_ almost 6 times higher than the positive control sodium lauryl sulphate (57.80 *μ*g/mL).

**Figure 3:**
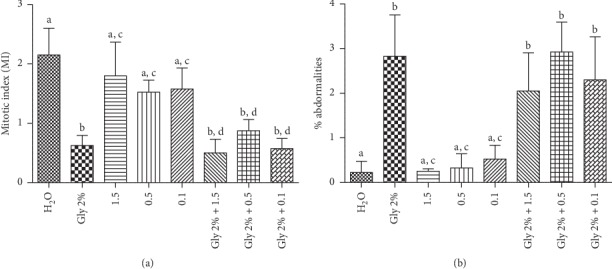
(a) Mitotic Index (MI) and (b) Percentage of Abnormalities (AN) for *C. nutans* leaf obtained in the *Allium cepa* assay. Different letters represent statistical differences according to the Tukey test (<0.001).

The genotoxicity test (Figure 3) did not indicate significant difference in MI of leaf concentrations tested (Figure 3(a)) in relation to the negative control, demonstrating that the extract did not interfere with the MI. Statistical analysis of the abnormalities showed that, in the three concentrations in which extracts (0.1, 0.5, 1.5 mg/mL) were tested, there was a significant difference from the glyphosate positive control (Figure 3(b)), demonstrating that the sample did not show genotoxicity to *A. cepa* roots. However, in the analysis of damage prevention, it is possible to observe in the three leaf concentrations that are statistically equal to the positive control, indicating, for these concentrations, that the extract was not able to prevent glyphosate damage.

### 3.5. *Drosophila melanogaster* Assays

#### 3.5.1. Survival

**Figure 4:**
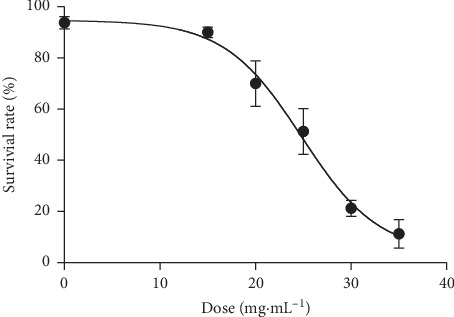
LC_50_ (24.83 mg/mL) of the hydromethanolic *C. nutans* extract leaf response among adult *Drosophila melanogaster* exposed to PQ in the feed. The results expressed in percentage of survivor flies presented correspond to the concentrations of the 15, 20, 25, 30, *e* 35 mg/mL. The test was effected in quadruplicate. *R*^2^ = 0.8997.

The determination of the lethal concentration of *C. nutans* leaf extract, that kills 50% (LC_50_) of a test population of the common fruit fly, *Drosophila melanogaster (D. melanogaster)*, which is the benchmark concentration for toxicity studies. In this study, the LC_50_ of *C. nutans* leaf extract obtained was 24.83 mg/mL five days after exposure at the concentrations tested (Figure 4). This result demonstrated that the extract leaf has a low degree of toxicity and suggest that can be used in pharmacological studies future.

#### 3.5.2. Paraquat Exposure and *C. nutans* Leaf Extract Treatment

**Figure 5:**
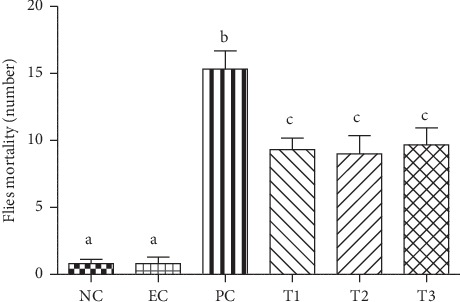
PQ poisoning control trial represented by mortality (number of dead individuals) per group. NC: Negative Control (1% sucrose); EC: Extract Control (10 mg/mL extract leaf); PC: Positive Control (3 mM PQ); and treatment: (*T*1 = 3 mM PQ + 1 mg/mL extract; *T*2 = 3 mM PQ + 5 mg/mL extract; *T*3 = 3 mM PQ + 10 mg/mL extract). Different letters represent statistical differences according to Tukey's test (*p* < 0.0001).

Paraquat (PQ, 1,10-dimethyl-4,40-bipyridinium dichloride) is commonly used in the laboratory to generate oxidative stress. *In vivo*, PQ radical reacts with oxygen to generate superoxide anion, a reactive oxygen species (ROS). Posteriorly, excess of ROS and depletion of reducing agents lead to oxidative stress, resulting in ROS damages of lipids, proteins, and DNA, potentially leading to cell death [38]. In the present study, flies were exposed to treatments for 4 days exposure to PQ (3 mM) and *C. nutans* extract leaf at the three concentrations (1, 5, and 10 mg/mL). Results demonstrated that the extract leaf tested show a reduced fly mortality by approximately 40% in relation to PQ (Figure 5), demonstrating that the extract is able to prevent PQ damage.

#### 3.5.3. Negative Geotaxis Assay

The use of *C. nutans* leaf extract to remedy the effect caused to the locomotor system (neurotoxic effect) by the use of the herbicide PQ was evaluated using *D. melanogaster* as a model. The use of PQ as a stressor is well-documented in the literature, affecting the nervous system and, consequently, the locomotor system [39].

**Figure 6:**
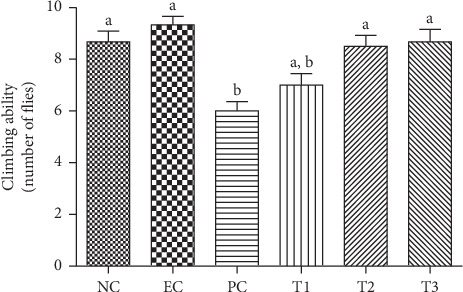
Effect of *C. nutans* extract on negative geotaxis (10 flies per replicate) in flies exposed to paraquat for 4 days (*n* = 3). NC: Negative Control (1% sucrose); EC: Extract Control (10 mg/mL extract leaf); PC: Positive Control (3 mM PQ); and treatment: (*T*1 = 3 mM PQ + 1 mg/mL extract; *T*2 = 3 mM PQ + 5 mg/mL extract; *T*3 = 3 mM PQ + 10 mg/mL extract). Different letters represent statistical differences according to Tukey's test (*p* < 0.0001).

Three concentrations of *C. nutans* leaf extract (1, 5 and 10 mg mL^−1^) and their effects on the PQ neurostressor (3 mM) were used. Negative geotaxis assay result (Figure 6) indicates a dose-dependent behavior on *C. nutans* extract on locomotor activity. An increased neuroprotective effect is observed with increasing concentration of the extract. This behavior is first observed in *C.nutans* species. A similar effect was observed by our research group [40] using Bougainvillea leaf extract and PQ.

## 4. Discussion

Phytochemical screening for *C. nutans* leaves revealed the presence of polyphenols, flavonoids, alkaloids, coumarins, condensed tannins, quaternary bases, and free steroids. No studies have been observed in the literature to prove the presence of all these phytoconstituents, neither at genus, and only coumarins was described by Truiti and Sarragioto [18] for the species. Positive results for the presence of flavonoids, coumarins, tannins, and steroids were found for the crude extract of *Tithonia diversifolia* leaves and negative for saponins [41], similar to the results obtained by us as *C. nutans* belong to the same family.

Chromatographic analysis (HPLC-DAD-MS) shows that the composition of the methanolic extract of *C. nutans* leaves contains phenolic compounds, a large number of substances, from single molecules to others with a high degree of polymerization [42] and present in vegetables in free form or linked to sugars (glycosides) and proteins [43]. Phenolic compounds are divided into three major groups: flavonoids and derivatives, phenolic acids (benzoic acids, cinnamic acids, and their derivatives), and coumarins [44]. They have a wide variety of substances characterized by the presence of one or more aromatic rings attached to at least one hydroxyl radical and/or other substitutes. It can be divided according to the number of phenolic rings and the structures to which they are attached [45]. Results (Table 1) revealed that phenolic acids were the most abundant polyphenols detected for *C. nutans* leaf extract as quinic acid, 4-phenylbutiric acid, isoferulic acid, 5-hydroxyanthranilic acid, 3-hydroxybenzoic acid, and arbutin. According to the literature, quinic acid is a potent antioxidant [46], hepatoprotective [47], and can be used to combat prostate cancer [48]. Studies have shown that the use of 4-phenylbutirc acid in the regulation of oxidative stress attenuated cell damage and acted as a cytoprotector and may be related to inhibition of oxidative stress [49]. Yang et al. [50] claim that the ability of ethanolic propolis extracts to act as antioxidants and to eliminate free radicals is due to the presence of phenolic acids, including isofeluric acid. Bakr [51], studying *Artemisia judaica*, observed a powerful free radical scavenging activity compared to ascorbic acid justifying this activity to the high content of phenolic and flavonoid acids, among the phenolic acids is the presence of isofeluric acid. Arbutin is a phenolic glycoside of plants, well known for medicinal value and widely used in cosmetics. Studies have proven its antifungal and antioxidant activity [52] and effectively used to treat urinary tract infections [53]. 3-hydroxybenzoic acid, not a secondary plant metabolite, was found in small quantities in green tea samples by Gruz et al. [54], suggesting that possible contamination may have occurred by microorganisms found in soil and/or animal excreta. Similar result may have occurred in our study. 5-hydroxyanthranilic acid is an acid that contains portions of coumaric, caffeic, and ferulic acid, being found in avenanthramides organic molecules extracted from oats, widely used in cosmetics [55]. The qualitative analysis of the main compounds present in the extract of *C. nutans* leaves may be useful to clarify the relationships between the content of phenolic compounds and total flavonoids and their antioxidant capacity.

Flavonoids, tannins, and phenolic substances are constituents of plants with potential antioxidant activity, mainly because they act as free radical scavengers [56]. Regarding the presence of these metabolites, no quantification studies were found in the literature, neither for species nor for *Chaptalia* genus. The results obtained for tannins, flavonoids, and polyphenols in *C. nutans* species (Table 2A) may justify their popular use as a laxative and bicheal internally and in topical preparations under injury, trauma, and hemorrhage [12, 14], due to the pharmacological potential of many species of the Asteraceae family to be related to the high tannin and flavonoid concentrations [57], since many of their medicinal properties are often attributed to these secondary metabolites.

Pretti et al. [41] found values for the flavonoids and polyphenols content for *T. diversifolia leaves*, as verified in our study, differing only in the tannin content, which was found in greater quantity. Nalewajko-Sieliwoniuk et al. [58] observed a high content of phenolic compounds in the methanolic extracts of the shoots, mainly in the *Erigeron acris* leaves, a species belonging to the Asteraceae family. Additionally, Johari and Khong [59] observed for *P. bleo* methanolic extract a content of phenolic compounds (40.82 mg GAE/g) close to that found for *C. nutans* leaves (30.17 mg GAE/g). The authors justify the high antioxidant capacity of the species for having a high content of phenolic compounds.

Flavonoids are a class of polyphenols that are abundantly present among plant secondary metabolites. These compounds have great pharmacological importance, resulting from some properties of this class as anticarcinogenic, anti-inflammatory, antiulcerogenic, antiviral [60], antimutagenic, antioxidant and antimicrobial action [61], antiallergic, antihepatotoxic, antiosteoporotic, and even antitumor [62].

Nowadays, there is much interest in tracking the antioxidant activity of plant or food extracts to investigate possible medicinal properties [63]. For the evaluation of antioxidant activity, one of the methods used was the DPPH (2,2-diphenyl-1-picrylhydraza) method, one of the most effective, simple, and reliable *in vitro* methods that has the ability to sequester free radicals. DPPH is stable violet organic nitrogen radical and has a maximum absorption in the range of 515–520 nm [64], where the lower the IC_50_, the higher the antioxidant activity of the material. For the DPPH method, the extract of *C. nutans* leaves presented an intermediate value (Table 2B), indicating moderately antioxidant activity of the extract. For the *U. baccifera* species, a moderate intensity activity has been described in this [65] due to the high IC_50_ reported (118.31 *μ*g/mL). Choi et al. [26] reinforce that due to the complexity of the chemicals present in crude extracts, it is necessary to evaluate the antioxidant capacity of the plant by at least two methods.

In this sense, another method for evaluating antioxidant activity used was the Ferric Reducing Antioxidant Power (FRAP) method; it is being widely used among antioxidant analyses as it is an analysis that involves the reduction of Fe^3+^ to Fe^2+^, changing its coloration to blue in the presence of antioxidant substances [27]. The reducing power obtained for the species under study was 379.98 ± 39.25 *μ*g/mL (Table 2B), demonstrating a promising antioxidant capacity, since for studies with species of the same family were found similar values for the leaves of *T. diversifolia* (334 *μ*g/mL), using the same methodology, exhibiting antioxidant activity [41]. The same authors state that the reducing activity of the extract may occur due to the high content of polyphenols, flavonoids, and tannins.

To evaluate the oxidative stress parameters, the effect of the crude extract of *C. nutans* leaves on lipoperoxidation, protein oxidation, and the neutralizing capacity of oxygen radicals was measured through the levels of TBARS, carbonyl protein, and DCFH. Results demonstrate that the extract has the ability to protect the cell from protein and lipid damage, as well as the inhibition of oxygen-derived radicals at the three concentrations tested, 0.1, 1, and 10 mg/mL (Figures 1(a)–1(c)). Bahramikia et al. [66] observed in the crude (ethanol) extracts of *T. polium* and *C. rotunduns* the significant effect of the extracts on protein oxidation inhibition and lipid peroxidation levels compared with the control. Esteves and Cava [67] affirm that there is evidence that protein oxidation may be associated with lipid oxidation.

Figure 1(a) shows the effect of *C. nutans* extract on lipid peroxidation level according to TBARS assay without the presence of an inducing agent. The presence of this agent generates reactive oxygen and nitrogen species (RONS), which among free radicals are the main oxidizing agents. In this sense, another TBARS test was performed using ferrous sulfate as an inducing agent. Result indicates the great effect of the extract to 1000 *μ*g/mL, reversing the induced damage (Figure 5) and thus avoiding lipid peroxidation, which can be defined as a set of biochemical events resulting from the action of radicals on cell membrane unsaturated lipids, leading to destruction of their structure, failure of metabolite exchange mechanisms, and cell death [68, 69]. Oxidative stress results from an imbalance between the generation of oxidizing compounds and the action of antioxidant defense systems. Antioxidant defense mechanisms aim to limit RONS levels and control the occurrence of cell damage [70, 71].

Budni et al. [72], using a similar methodology, verified for the crude extract of *Tabebuia heptaphylla* leaves a reduction in ferrous sulphate-induced lipid peroxidation in three different concentrations tested. The same behavior was observed to ethanolic extract of *Mikania glomerata* leaves, where it was able to reduce induced lipid peroxidation [73]. Both studies cited confirm the antioxidant capacity of the species, which corroborates our studies. The high *in vitro* antioxidant capacity of the crude extract of *C. nutans* leaves is due to the presence of active substances such as phenolic compounds and the presence of coumarins [73], which are antioxidant compounds. These results are in agreement with the literature that among the several classes of naturally occurring antioxidant substances, phenolic compounds in plants have received attention in recent years, as it covers a range of substances, from simple molecules to those with a high degree of polymerization, proving the antioxidant activity of phenolic acids in inhibiting lipid peroxidation [74, 75].

The crude extract of the species studied was able to significantly reduce the oxidation of DCFH compared to the basal group (Figure 1(c)), demonstrating pronounced antioxidant activity in the three concentrations tested, which indicates that the extract of *C. nutans* leaves has the ability to neutralize different sources of RONS.

Similar results were observed by de Brum et al. [76] for the crude extract of *V. Megapotamica* leaves, which is considered an antioxidant species. The effect of reduction on oxidative stress observed in *C. nutans* can be attributed to the phytochemical composition of the extract. It is suggested that this effect is due to the presence of polyphenols, flavonoids, and tannins found, contributing to the antioxidant activity and also due to the demonstrated ability to eliminate RONS. Fabri et al. [64] and Paula et al. [77] also observed antioxidant activities in other Asteraceae species confirming the widespread popular use of this family in pathologies related to RONS production.

The ability of phenolic compounds to act as antioxidants depends on intrinsic factors, such as their own chemical structure and the intensity of oxidative reactions [78]. The significant antioxidant activity demonstrated by methanolic extract of *C. nutans* leaves has a positive relationship with the presence of phenolic compounds. Studies have shown that the antioxidant capacity of crude *P. bleo* leaf extract is highly associated with the total flavonoid content and total phenolic compounds present in the plant [59]. Corroborating our results for *C. nutans*, Taskin et al. [79] demonstrated that *A. grandifolia* is rich in flavonoids and phenolic acids and can be a good natural source of antioxidant.

In the last two decades, studies have been conducted with medicinal plants in different countries to prove their effectiveness as antimicrobial agents. Maddila and Hemalatha [80] report that global antibacterial resistance is becoming a growing public health problem, as bacterial resistance to most available antibacterial has been reported. The pharmaceutical industry and new biotechnology companies are intensifying efforts to discover new antibacterial in attempts to overcome bacterial resistance [80]. In this sense, the crude extract of *C. nutans* leaves and their fractions (hexane, chloroform, ethyl acetate, and butanol) was tested, but only the hexane extract of the leaves was promising against the microorganisms (Table 3). Truiti et al. [19] tested the crude extract and root fractions of *C. nutans* against *S. aureus, E. coli*, *and P. aeruginosa* being the extract considered susceptible to *S. aureus* and resistant to *E. coli* and *P. aeruginosa*. Antimicrobial activity for extracts of *C. nutans* leaves was previously reported by Heinrich et al. [81] where they observed action on *E. coli, B. subtilis*, *and M. luteus* and by Souza et al. [17] who reported action only for *B. subtilis* being resistant to the other microorganisms tested, including *E. coli*. Our results are in agreement with those of Truiti et al. [19] and Souza et al. [17] which show resistance of *E. coli* on leaf extract differing from the results of Heinrich et al. [81]. Such differences in the action of the extract on the same microorganism may be related to the different extraction techniques. The ability of leaf extract to inhibit the growth of microorganisms is caused by the presence of secondary metabolites in plant cells [82]. According to Janovik et al. [83], tannin-rich plants are used in folk medicine as antiseptic because the basis of their mechanism of action is the ability to precipitate proteins, forming a tannin-protein complex on damaged tissues preventing the development of microorganisms. . Thus, we can infer that the low tannin content in the extracts of *C. nutans* leaves is not sufficient for it to have a good antimicrobial potential.

The *Artemia salina* (*A. Salina*) toxicity test is a biological assay considered as one of the most widely used tools for preliminary toxicity assessment of plant extracts [84]. Extracts of plants with high toxicity against *A. salina* suggest high potential for biological activities, so it is very useful to use this bioassay in the direction of phytochemical studies in the search for bioactive substances [85]. In this sense, the LC_50_ (342.89 *μ*g/mL) found for the extract of *C. nutans* leaves (Table 4) suggests that it has moderate toxicity. According to NGUTA et al. [86], both organic extracts and aqueous extracts with LC_50_ values lower than 100 *μ*g/mL have high toxicity, LC_50_ between 100 and 500 *μ*g/mL have moderate toxicity, LC_50_ between 500 and 1000 *μ*g/mL have low toxicity, and LC_50_ above 1000 *μ*g/mL are considered nontoxic. Similar results were found for the methanolic extract of *Callicarpa candicans* (Verbenaceae) leaves with LC_50_ 383.9 *μ*g/mL which also antimicrobial activity [82] for the ethanolic extracts of the stem and leaves of *Dasyphyllun tomentosum* [87], *Neurolaena lobata* leaves [81], and *Mikania cordata* leaves [88] where they presented moderate toxicity and absence of toxicity using the *A. salina* model, which belong to the Asteraceae family and have antimicrobial and antitumor potential.

Due to its reliability and agreement with other genotoxicity assays, the *Allium cepa (A.cepa)* test system is generally employed for the preliminary evaluation of the genotoxicity of medicinal plants [35, 89]. The effects of medicinal plant infusions on the *A. cepa* cell cycle have been reported by several authors [90, 91, 92], which showed that the main effects that occur are mutagenicity and antimutagenicity, as well as increase and decrease of cell proliferation of root tips treated with different species of medicinal plants. The extract of the leaves of *C. nutans* did not inhibit the mitotic index and did not cause abnormalities in the concentrations were tested (Figure 2), thus showing no antiproliferative effect or genotoxic to *A. cepa* cells. Using the same methodology, Frescura et al. [93] found no genotoxic and antiproliferative action for the leaves and bark of *Luehea divaricata*, and the same behavior was observed for extract of *Euphorbia hirta* [94], *Icacia trichantha* leaf extracts [95], and *Amaranthus spinosus* aqueous extracts [91]. Regarding the ability to prevent glyphosate damage, the species under study was not promising, but did not cause chromosomal anomalies, demonstrating that its popular use will not cause cellular damage. Thus, it can be said that the *A. salina* toxicity bioassay and the *A. cepa* genotoxicity assay were effective to obtain preliminary results regarding the toxic potential of *C. nutans*. Additionally, the fractionation of these extracts can help in their safety evaluation in order to confirm the safety of the use of this plant by the population.

As the first model to evaluate the toxicity of the extract was the LC_50_ assay with *A. salina*, it is a simple, fast, and less expensive model. It was demonstrated that the concentration required to kill half of the individuals was 6 times in relation to positive control concentration, demonstrating that the extract has low toxicity against this model. Thus, searching a more complex experimental model, *D. melanogaster* was used, which is an *in vivo* organism widely used as an experimental model, presenting a range of advantages such as short life cycle, low maintenance cost, and ease of handling. In addition to having its genome sequenced [96] and its well-studied CNS, it is composed of about 1000 neurons, making it a great model for evaluating plant extract toxicities, neurotoxicity, and neurodegenerative diseases [40, 97, 98].

Regarding the toxicity of *C. nutans* extract against *D. melanogaster*, it was found that until the concentration of 15 mg/mL the extract showed no toxicity and that the LC_50_ obtained was 24 mg/mL (Figure 4), distant values of the concentrations were used in the defense tests against oxidative stress. Brito Junior et al. [99] verified for crude extract of *Croton campestris* leaves a LC_50_ of 26.51 mg/mL after 4 days of treatment, approximately as found for *C. nutans* in 5 days of treatment, showing that the species under study is safe because it needs a very high concentration to be toxic, indicating that it may have pharmacological application of the extract in the future.

In this way, the effect of methanolic extract of *C. nutans* leaves on *D. melanogaster* poisoning and locomotor damage caused by PQ herbicide was tested, being widely used as stressor agent in behavioral and intoxication tests. In the last decade, the toxicity of PQ has been described after this herbicide is responsible for significant brain damage and death of individuals following acute exposure [100] being very useful for evaluating neuroprotective compounds against movement disorders and PQ-induced neurodegeneration [101]. The extract of *C. nutans* leaves proved to be effective in reversing the action of herbicide on fly intoxication (Figure 5), reducing mortality by 40% and the ability to reverse locomotor damage (Figure 6) induced by PQ, since a better fly performance was observed in climbing at higher concentrations, indicating that the extract has the ability to protect against mortality and brain damage induced by PQ. Soares et al. [40] observed similar results where the action of *Bougainvillea glabra* extract was able to reduce the mortality rate and neurotoxicity of flies when used concomitantly with PQ. Also, it was found that *Decalepis hamiltonii* root extracts were able to protect flies from mortality and PQ-induced locomotor impairment [39]. Different results for *Croton campestris* hydroalcolic extract were observed and it was toxic when administered concomitantly with PQ, increasing the mortality rate, as well as changing the locomotor behavior of flies [99].

Exposure to PQ herbicide is recognized as a major risk factor for the manifestation of neurodegenerative diseases. PQ neurotoxicity is attributed to its cyclic redox effect that generates a significant amount of reactive oxygen species (ROS) leading to oxidative stress [40]. Results show that the three concentrations used to prevent PQ damage were effective in reducing fly mortality (Figure 5) and improving locomotor capacity (Figure 6), as it is known that these effects caused by PQ come from oxidative stress, several studies that consider oxidative stress as the main mechanism of PQ-induced toxicity [96]. These results show that the good *in vitro* antioxidant activity may be related to this protective effect of PQ damage in *in vivo* models. This is justified by the presence of antioxidant compounds identified in the extract, since phenolic compounds present in plants have redox properties, which act as antioxidants [59], and their protective effects against PQ alone have been proven [39].

According to the results of cytotoxic, neurotoxic, antioxidant capacity, and HPLC-DAD-MS assays, methanolic extract of *C. nutans* leaves can be used as a source of natural antioxidant, however, additional *in vitro* studies, as cell cuttings and *in vivo* with rodent and aquatic species. In addition, further investigation is needed to reveal whether the extract can reduce other dysfunctional factors involved in the neurodegeneration process.

## 5. Conclusion

This study demonstrates in an unprecedented way that the crude extract of *C. nutans* leaves is rich in phenolic compounds and flavonoids and has the capacity to neutralize different sources of ROS, as well as presents low toxicity and absence of cyto- and genotoxicity. Thus, it is suggested that there is a synergism between the chemical composition of the extract, especially phenolic compounds, with the high antioxidant capacity demonstrated through different analysis techniques and the neuroprotective action of the extract where it was able to protect against oxidative damage and locomotor in *D. melanogaster* caused by PQ. Therefore, our results open the way for the possible development of natural antioxidants after further studies for the isolation of compounds and more specific investigations to elucidate the mechanisms of action of the extract at more complex cellular and organism levels, as well as its pharmacological evaluation.

## Figures and Tables

**Table 1:** Compounds identified in extracts of *Chaptalia nutans* by HPLC-DAD-MS.

Sample	Compound	Rt (min)	(M-H) (*m*/*z*)	Fragment ions in MS/MS (*m*/*z*)	Reference
LV	Quinic acid	2.4–2.9	191	103, 133	Bouhafsoun et al., 2018
	4-Phenylbutyric acid	3.0–3.4	147		Kato-Noguchi, 2008
	Isoferulic acid	14.2–15.5	193	134	Aghraz et al., 2018; Bakr, 2014; Yang, 2011
	5-Hydroxyanthranilic acid	3.5–4.0	107	—	Magee et al., 2007
	3-Hydroxybenzoic acid	2.6	93	—	Gruz et al., 2008
	Arbutin	14.4–14.6	108	—	Ekiert et al., 2012; Urbanska et al., 2014

Rt = retention time; (M-H)^−^ (*m*/*z*) = molecular ion peak in negative mode.

**Table 2:** A: Polyphenols, flavonoids, and condensed tannins contents. B: IC_50_ results (DPPH) (*μ*g/mL ± SD). Ferric reducing antioxidant power (FRAP) assay (*μ*M FeSO_4_/mg sample). Results for crude extract (CE) of C. *nutans* leaf.

	CE ± SD *C*. *nutans* leaf
A	
Polyphenols (mg/g ± SD)	30.17 ± 1.44
Flavonoids (mg/g ± SD)	21.64 ± 0.66
Condensed tannins (mg/g ± SD)	9.58 ± 0.99

B	
DPPH IC_50_ (*μ*g/mL ± SD)	345.41 ± 5.35
FRAP assay (*μ*M FeSO_4_/mg sample)	379.98 ± 39.25

CE: crude extract; SD: standard deviation.

**Table 3:** Results of antimicrobial activity for hexane fraction of C. *nutans* leaf.

Bacterium/fungi	Leaf
*E.coli*	>2000 *μ*g/mL
*S. epidermidis*	1000 *μ*g/mL
*C. tropicalis*	1000 *μ*g/mL
*C. glabrata*	1000 *μ*g/mL
*C.krusei*	1000 *μ*g·mL^−1^

**Table 4:** Lethal concentration for 50% of *Artemia salina* nauplii (LC_50_) and confidence interval obtained for *C. nutans* leaf and the positive control.

	LC_50_ (*μ*g/mL) >	Confidence interval (*μ*g/mL)
Leaf	342.89	207.06–478.75
Sodium lauryl sulfate	57.80	56.10–59.40

## Data Availability

The data used to support the findings of this study are available from the corresponding author upon request.

## References

[1] Yadav V. K. (2018). Phytochemical and pharmacognostical studies of *Blumea lacera*. *International Journal of Green Pharmacy*.

[2] Tian X.-R., Feng J.-T., Ma Z.-Q. (2014). Three new glycosides from the whole plant of *Clematis lasiandra* Maxim and their cytotoxicity. *Phytochemistry Letters*.

[3] Egamberdieva D., Mamedov N., Ovidi E., Tiezzi A., Craker L. (2016). Phytochemical and pharmacological properties of medicinal plants from Uzbekistan: a review. *Journal of Medicinally Active Plants*.

[4] Pradeepa S., Subramanian S., Kaviyarasan V. (2014). Evaluation of antimicorbial activity of *Pithecellobium Dulce* pod pulp extract. *Asian Journal of Pharmaceutical and Clinical Research*.

[5] Ayres M. C. C., Chaves M. H., Rinaldo D., Vilegas W., Vieira Júnior G. M. (2009). Constituintes químicos e atividade antioxidante de extratos das folhas de *Terminalia fagifolia* Mart. et Zucc. *Química Nova*.

[6] Ulewicz-Magulska B., Wesolowski M. (2019). Total phenolic contents and antioxidant potential of herbs used for medical and culinary purposes. *Plant Foods for Human Nutrition*.

[7] Vasconcelos S. M. L., Goulart M. O. F., Moura J. B. F., Manfredini V., Benfato M. S., Kubota L. T. (2007). Espécies reativas de oxigênio e de nitrogênio, antioxidantes e marcadores de dano. *Química Nova*.

[8] Clarke J. H. R., Rates S. M. K., Bridi R. (2007). Um alerta sobre o uso de produtos de origem vegetal na gravidez. *Infarma*.

[9] Nesom G. L. (1995). Revision of *Chaptalia* (Asteraceae: mutisieae) from North America and continental Central America. *Phytologia*.

[10] Kissmann K. G., Groth D. (1997). *Plantas Infestantes e Nocivas*.

[11] Lorenzi H., Matos F. J. A. (2002). *Plantas Medicinais no Brasil: Nativas e Exóticas Cultivadas*.

[12] Cabrera A. L., Klein R. M., Reitz R. (1973). Compostas—tribo mutisieae. *Flora Ilustrada Catarinense*.

[13] Corrêa M. P. (1984). Dicionário das plantas úteis do Brasil e das exóticas cultivadas.

[14] Pasini E., Katinas L., Ritter M. R. (2014). O gênero chaptalia (Asteraceae, mutisieae) no Rio Grande do Sul, Brasil. *Rodriguésia*.

[15] Empinotti C. B., Duarte M. R. (2006). Caracteres anatômicos de arnica-do-campo: *Chaptalia nutans*. *Acta Farmacéutica Bonaerense*.

[16] Badilla B., Mora G., Poveda L. J. (2000). Anti-inflammatory activity of aqueous extracts of five Costa Rican medicinal plants in Sprague-Dawley rats. *Revista de Biologia Tropical*.

[17] Souza G. C., Haas A. P. S., von Poser G. L., Schapoval E. E. S., Elisabetsky E. (2004). Ethnopharmacological studies of antimicrobial remedies in the south of Brazil. *Journal of Ethnopharmacology*.

[18] Truiti M. D. C. T., Sarragiotto M. H. (1998). Three 5-methylcoumarins from *Chaptalia nutans*. *Phytochemistry*.

[19] Truiti M. C. T., Sarragiotto M. H., Abreu Filho B. A., Nakamura C. V., Dias Filho B. P. (2003). In vitro antibacterial activity of a 7-O-beta-Dglucopyranosyl- nutanocoumarin from *Chaptalia nutans* (Asteraceae). *Memórias Do Instituto Oswaldo Cruz*.

[20] Osafo N., Mensah K., Kofi Yeboah O. (2017). Phytochemical and pharmacological review of *Cryptolepis sanguinolenta* (lindl.) schlechter. *Advances in Pharmacological Sciences*.

[21] Matos F. J. A. (2009). *Introdução a Fitoquímica Experimental*.

[22] Chandra S., Gonzalez de Mejia E. (2004). Polyphenolic compounds, antioxidant capacity, and quinone reductase activity of an aqueous extract of ardisia compressain comparison to mate (Ilex paraguariensis) and green (Camellia sinensis) teas. *Journal of Agricultural and Food Chemistry*.

[23] Woisky R. G., Salatino A. (1998). Analysis of propolis: some parameters and procedures for chemical quality control. *Journal of Apicultural Research*.

[24] Morrison I., Asiedu E. A., Stuchbury T., Powell A. A. (1995). Determination of lignin and tannin contents of Cowpea seed coats. *Annals of Botany*.

[25] Vieira R. F., Grayer R. J., Paton A., Simon J. E. (2001). Genetic diversity of *Ocimum gratissimum* L. based on volatile oil constituents, flavonoids and RAPD markers. *Biochemical Systematics and Ecology*.

[26] Choi C. W., Kim S. C., Hwang S. S. (2002). Antioxidant activity and free radical scavenging capacity between Korean medicinal plants and flavonoids by assay-guided comparison. *Plant Science*.

[27] Rufino M. S. M., Alves R. E., Brito E. S. (2006). *Metodologia Científica: Determinação da Atividade Antioxidante Total em Frutas Pelo Método de Redução do Ferro (FRAP)*.

[28] Ohkawa H., Ohishi N., Yagi K. (1979). Assay for lipid peroxides in animal tissues by thiobarbituric acid reaction. *Analytical Biochemistry*.

[29] Vyncke W. (1970). Direct determination of the thiobarbituric acid value in trichloracetic acid extracts of fish as a measure of oxidative rancidity. *Fette, Seifen, Anstrichmittel*.

[30] Levine R. L., Garland D., Oliver C. N. (1990). Determination of carbonyl content in oxidatively modified proteins. *Oxygen Radicals in Biological Systems Part B: Oxygen Radicals and Antioxidants*.

[31] Myrhe O., Andersen J. M., Aarnes H., Fonnum F. (2003). Evaluation of the probes 2′,7′-dichlorofluorescin diacetate, luminol, and lucigenin as indicators of reactive species formation. *Biochemical Pharmacology*.

[32] CLSI—Clinical Laboratory Standards Institute (2012). *Methods for Dilution Antimicrobial Susceptibility Tests for Bacteria that Grow Aerobically*.

[33] CLSI—Clinical and Laboratory Standards Institute (2008). *Reference Method for Broth Dilution Antifungal Susceptibility Testing Yeasts*.

[34] Silva L. L., Heldwein C. G., Reetz L. G. B., Hörner R., Mallmann C. A., Heinzmann B. M. (2010). Composição química, atividade antibacteriana in vitro e toxicidade em *Artemia salina* do óleo essencial das inflorescências de *Ocimum gratissimum* L., Lamiaceae. *Revista Brasileira de Farmacognosia*.

[35] Tedesco S. B., Laughinghouse H. D., Srivastava J. K. (2012). Bioindicator of genotoxicity: the *Allium cepa* test. *Environmental Contamination*.

[36] Guerra M., Souza M. J. (2002). *Como Observar Cromossomos: um Guia de Té cnica em Citogenética Vegetal, Animal e Humana*.

[37] Soares J. J., Gonçalves M. B., Gayer M. C. (2017). Continuous liquid feeding: new method to study pesticides toxicity in *Drosophila melanogaster*. *Analytical Biochemistry*.

[38] Rzezniczak T. Z., Douglas L. A., Watterson J. H., Merritt T. J. S. (2011). Paraquat administration in *Drosophila* for use in metabolic studies of oxidative stress. *Analytical Biochemistry*.

[39] Jahromi S. R., Haddadi M., Shivanandappa T., Ramesh S. R. (2013). Neuroprotective effect of *Decalepis hamiltonii* in paraquat-induced neurotoxicity in *Drosophila melanogaster*: biochemical and behavioral evidences. *Neurochemical Research*.

[40] Soares J. J., Rodrigues D. T., Gonçalves M. B. (2017). Paraquat exposure-induced Parkinson’s disease-like symptoms and oxidative stress in *Drosophila melanogaster*: neuroprotective effect of *Bougainvillea glabra* Choisy. *Biomedicine & Pharmacotherapy*.

[41] Pretti I. R., Luz A. C. D., Jamal C. M., Batitucci M. D. C. P. (2018). Variation of biochemical and antioxidant activity with respect to the phenological stage of *Tithonia diversifolia* Hemsl. (Asteraceae) populations. *Industrial Crops and Products*.

[42] Bravo L. (1998). Polyphenols: chemistry, dietary sources, metabolism and nutrition significance. *Nutrition Reviews*.

[43] Croft K. D. (1998). The chemistry and biological effects of flavonoids and phenolic acidsa. *Annals of the New York Academy of Sciences*.

[44] Soares S. E. (2002). Ácidos fenólicos como antioxidantes. *Revista de Nutrição*.

[45] Oliveira D. M. D., Bastos D. H. M. (2011). Biodisponibilidade de ácidos fenólicos. *Química Nova*.

[46] Pero R. W., Lund H., Leanderson T. (2009). Antioxidant metabolism induced by quinic acid increased urinary excretion of tryptophan and nicotinamide. *Phytotherapy Research*.

[47] Xiang T., Xiong Q.-B., Ketut A. I. (2001). Studies on the hepatocyte protective activity and the structure-activity relationships of quinic acid and caffeic acid derivatives from the flower buds of *Lonicera bournei*. *Planta Medica*.

[48] Inbathamizh L., Padmini E. (2013). Quinic acid as a potent drug candidate for prostate cancer—a comparative pharmacokinetic approach. *Asian Journal of Pharmaceutical and Clinical Research*.

[49] Wei H., Kim S.-J., Zhang Z. (2008). ER and oxidative stresses are common mediators of apoptosis in both neurodegenerative and non-neurodegenerative lysosomal storage disorders and are alleviated by chemical chaperones. *Human Molecular Genetics*.

[50] Yang H., Dong Y., Du H., Shi H., Peng Y., Li X. (2011). Antioxidant compounds from propolis collected in Anhui, China. *Molecules*.

[51] Bakr R. O. (2015). Microscopical and phytochemical investigation of Egyptian *Artemisia judaica* L. var. sinaitica tackholm and its free radical scavenging activity. *International Journal of Pharmacognosy and Phytochemical Research*.

[52] Azadbakht M., Marston A., Hostettmann K., Ramezani M., Jahromi Maghddam M. (2004). Biological activity of leaf extract and phenologlycoside arbutin of *Pyrus boisseriana* Buhse. *Journal of Medicinal Plants*.

[53] Quintus J., Kovar K.-A., Link P., Hamacher H. (2005). Urinary excretion of arbutin metabolites after oral administration of bearberry leaf extracts. *Planta Medica*.

[54] Gruz J., Novák O., Strnad M. (2008). Rapid analysis of phenolic acids in beverages by UPLC-MS/MS. *Food Chemistry*.

[55] Magee L., Liebel F., Southall M. (2007). Compositions for inhibiting or reducing inflammation of skin.

[56] Simões C. M. O., Schenkel E. P., Gosmann G., Mello J. C. P., Mentz L. A., Petrovick P. R. (2010). *Farmacognosia da Planta ao Medicamento*.

[57] Santos S. C., Costa W. F., Ribeiro J. P. (2002). Tannin composition of barbatimão species. *Fitoterapia*.

[58] Nalewajko-Sieliwoniuk E., Pliszko A., Nazaruk J., Barszczewska E., Pukszta W. (2019). Comparative analysis of phenolic compounds in four taxa of *Erigeron acris* s. l. (Asteraceae). *Biologia*.

[59] Johari M. A., Khong H. Y. (2019). Total phenolic content and antioxidant and antibacterial activities of *Pereskia bleo*. *Advances in Pharmacological Sciences*.

[60] Granato E. M., Granato M. M., Gerenutti M. M., Silva G., Ferraz H. O., Vila M. M. D. C. (2013). Prospecção fitoquimica da espécie vegetal Trixis antimenorrhoea (Schrank) Kuntze. *Revista Brasileira de Farmácia*.

[61] Valdés L., Cuervo A., Salazar N., Ruas-Madiedo P., Gueimonde M., González S. (2015). The relationship between phenolic compounds from diet and microbiota: impact on human health. *Food & Function*.

[62] Omes S. V. F., Nogueira P. C. L., Morae S. V. R. S. (2011). Aspectos químicos e biológicos do gênero Lippia enfatizando Lippia gracilis Schauer. *Eclética Química Journal*.

[63] Ghani M. A., Barril C., Bedgood D. R., Prenzler P. D. (2019). Development of a method suitable for high-throughput screening to measure antioxidant activity in a linoleic acid emulsion. *Antioxidants*.

[64] Fabri R. L., Nogueira M. S., Dutra L. B., Bouzada M. L. M., Scio E. (2011). Potencial antioxidante e antimicrobiano de espécies da família Asteraceae. *Revista Brasileira de Plantas Medicinais*.

[65] Gindri A. L., de Souza L. B., Cruz R. C., Boligon A. A., Machado M. M., Athayde M. L. (2014). Genotoxic evaluation, secondary metabolites and antioxidant capacity of leaves and roots of *Urera baccifera* Gaudich (Urticaceae). *Natural Product Research*.

[66] Bahramikia S., Ardestani A., Yazdanparast R. (2009). Protective effects of four Iranian medicinal plants against free radical-mediated protein oxidation. *Food Chemistry*.

[67] Estévez M., Cava R. (2006). Effectiveness of rosemary essential oil as an inhibitor of lipid and protein oxidation: contradictory effects in different types of frankfurters. *Meat Science*.

[68] Benzie I. F. F. (1996). Lipid peroxidation: a review of causes, consequences, measurement and dietary influences. *International Journal of Food Sciences and Nutrition*.

[69] Lima É. S., Abdalla D. S. P. (2001). Peroxidação lipídica: mecanismos e avaliação em amostras biológicas. *Brazilian Journal of Pharmaceutical Sciences*.

[70] Barbosa K. B. F., Costa N. M. B., Alfenas R. D. C. G., de Paula S. O., Minim V. P. R., Bressan J. (2010). Estresse oxidativo: conceito, implicações e fatores modulatórios. *Revista de Nutrição*.

[71] Dias V., Junn E., Mouradian M. M. (2013). The role of oxidative stress in Parkinson’s disease. *Journal of Parkinson’s Disease*.

[72] Budni P., Petronilho F. C., Citadini-Zanette V. (2007). Estudos preliminares da atividade antioxidante do extrato hidroetanólico de folhas jovens e adultas de *Tabebuia heptaphylla* (Vell.) Toledo (ipê-roxo). *Latin American Journal of Pharmacy*.

[73] Santana L. C. L. R., Silva O. A., Brito M. R. M. (2013). Avaliação do potencial antioxidante, atividade antimicrobiana e antihelmíntica do extrato etanólico padronizado das folhas de *Mikania glomerata* Sprengel. *Revista Brasileira de Farmácia*.

[74] Azevedo R. R. D. S., Almeida V. G. A., Silva E. M. F. (2011). Potencial antioxidante e antibacteriano do extrato etanólico de plantas usadas como chás. *Revista Semente*.

[75] Santi M. M., Sanches F. S., Silva J. F. M., Santos P. M. L. (2014). Determinação do perfil fitoquímico de extrato com atividade antioxidante da espécie medicinal Cordia verbenacea DC. por HPLC-DAD. *Revista Brasileira de Plantas Medicinais*.

[76] de Brum T., Zadra M., Piana M. (2013). HPLC analysis of phenolics compounds and antioxidant capacity of leaves of *Vitex megapotamica* (Sprengel) Moldenke. *Molecules*.

[77] Paula C. S., Canteli V. C. D., Verdam M. C. S. (2014). Atividade antioxidante e toxicidade preliminar do extrato e frações obtidas das folhas e cascas do caule de *Dasyphyllum tomentosum* (Spreng.) Cabrera. *Revista Brasileira de Plantas Medicinais*.

[78] Lund M. N., Heinonen M., Baron C. P., Estévez M. (2011). Protein oxidation in muscle foods: a review. *Molecular Nutrition & Food Research*.

[79] Taşkin D., Alkaya D. B., Dölen E. (2018). Evaluation of antioxidant capacity and analysis of major phenolic compounds in *Achillea grandifolia* by HPLC-DAD with Q-TOF LC/MS confirmation. *Chiang Mai Journal of Science*.

[80] Maddila S., Hemalatha K. P. J. (2017). Phytochemical screening and *in vitro* antimicrobial properties of crude leaf extracts of *Wrightia tinctoria* R.Br. *International Journal of Current Microbiology and Applied Sciences*.

[81] Heinrich M., Kunhnt M., Wright C. W., Rimpler H., Phillipson J. D., Schandelmaier A. (1991). Lowland mixe Indian medicinal plants: parasitological and microbiological evaluation and initial phytochemical study of *Chaptalia nutans*. *Planta Medica*.

[82] Nurtjahja K., Kelana T. B., Suryanto D. (2013). Antimicrobial activity of endemic herbs from tangkahan conservation forest north sumatera to bacteria and yeast. *HAYATI Journal of Biosciences*.

[83] Janovik V., Boligon A. A., Feltrin A. C., Pereira D. F., Frohlich J. K., Athayde M. L. (2009). Doseamento de polifenóis, flavonóides e taninos no extrato bruto e frações de *Cariniana domestica* (Mart.) Miers. *Saúde (Santa Maria)*.

[84] Sarmento P. D. A., Ataíde T. D. R., Barbosa A. P. F., Araújo-Júnior J. X. D., Lúcio I. M. L., Bastos M. L. D. A. (2014). Evaluation of the extract of Zeyheria tuberculosa with a view to products for wound healing. *Revista Latino-Americana de Enfermagem*.

[85] Amarante C. B. D., Müller A. H., Póvoa M. M., Dolabela M. F. (2011). Estudo fitoquímico biomonitorado pelos ensaios de toxicidade frente à *Artemia salina* e de atividade antiplasmódica do caule de aninga (*Montrichardia linifera*). *Acta Amazonica*.

[86] Nguta J. M., Mbaria J. M., Gakuya D. W., Gathumbi P. K., Kabasa J. D., Kiama S. G. (2011). Biological screening of Kenyan medicinal plants using *Artemia salina* (Artemiidae). *Pharmacologyonline*.

[87] Mayorga P., Pérez K. R., Cruz S. M., Caceres A., Cáceres A. (2010). Comparison of bioassays using the anostracan crustaceans *Artemia salina* and *Thamnocephalus platyurus* for plant extract toxicity screening. *Revista Brasileira de Farmacognosia*.

[88] Nayeem A. A., Khatun A., Rahman M. S., Rahman M. (2011). Evaluation of phytochemical and pharmacological properties of *Mikania cordata* (Asteraceae) leaves. *Journal of Pharmacognosy and Phytotherapy*.

[89] Bagatini M. D., Silva A. C. F. D., Tedesco S. B. (2007). Uso do sistema teste de *Allium cepa* como bioindicador de genotoxicidade de infusões de plantas medicinais. *Revista Brasileira de Farmacognosia*.

[90] Chukwujekwu J. C., Van Staden J. (2014). Cytotoxic and genotoxic effects of water extract of *Distephanus angulifolius* on *Allium cepa* Linn. *South African Journal of Botany*.

[91] Prajitha V., Thoppil J. E. (2016). Genotoxic and antigenotoxic potential of the aqueous leaf extracts of *Amaranthus spinosus* Linn. using *Allium cepa* assay. *South African Journal of Botany*.

[92] Hister C. A. L., Pasqualli M., Trapp K. C. (2017). Atividade antiproliferativa e determinação dos compostos fenólicos de extratos aquosos de amoreira-preta (*Rubus sp*.) pelo sistema teste in vivo de *Allium cepa* L.. *Revista Brasileira de Biociências*.

[93] Frescura V. D.-S., Laughinghouse H. D., Tedesco S. B. (2012). Antiproliferative effect of the tree and medicinal species *Luehea divaricata* on the *Allium Cepa* cell cycle. *Caryologia*.

[94] Yuet Ping K., Darah I., Yusuf U. K., Yeng C., Sasidharan S. (2012). Genotoxicity of *Euphorbia hirta*: an *Allium cepa* assay. *Molecules*.

[95] Timothy O., Idu M., Olorunfemi D. I., Ovuakporie-Uvo O. (2014). Cytotoxic and genotoxic properties of leaf extract of *Icacina trichantha* Oliv. *South African Journal of Botany*.

[96] Kim S.-I., Jung J.-W., Ahn Y.-J., Restifo L. L., Kwon H.-W. (2011). Drosophila as a model system for studying lifespan and neuroprotective activities of plant-derived compounds. *Journal of Asia-Pacific Entomology*.

[97] Sudati J. H., Vieira F. A., Pavin S. S. (2013). *Valeriana officinalis* attenuates the rotenone-induced toxicity in *Drosophila melanogaster*. *NeuroToxicology*.

[98] Panchal K., Tiwari A. K. (2017). *Drosophila melanogaster* “a potential model organism” for identification of pharmacological properties of plants/plant-derived components. *Biomedicine & Pharmacotherapy*.

[99] Júnior F. E. B., Macedo G. E., Zemolin A. P. (2016). Oxidant effects and toxicity of croton campestrisin *Drosophila melanogaster*. *Pharmaceutical Biology*.

[100] Blanco-Ayala T., Andérica-Romero A. C., Pedraza-Chaverri J. (2014). New insights into antioxidant strategies against paraquat toxicity. *Free Radical Research*.

[101] Niveditha S., Ramesh S. R., Shivanandappa T. (2017). Paraquat-induced movement disorder in relation to oxidative stress-mediated neurodegeneration in the brain of *Drosophila melanogaster*. *Neurochemical Research*.

